# A 15 year-old-girl with persistent leg pain diagnosed as a giant cell tumor of the tibial diaphysis: A case report and review of the literature

**DOI:** 10.1016/j.ijscr.2022.107680

**Published:** 2022-09-20

**Authors:** Hasan Masud, Prashant Yadav, Sushmita Yadav, Mohammed kamal

**Affiliations:** a Department of Orthopedics, Sir Salimullah Medical College & Mitford hospital, Dhaka, Bangladesh; b Sir Salimullah Medical College & Mitford hospital, Dhaka, Bangladesh; c Jalalabad Ragib-Rabeya Medical College, Sylhet, Bangladesh; d Department of pathology, Bangabandhu Sheikh Mujib Medical University, Dhaka, Bangladesh

**Keywords:** Giant cell tumor of bone, Oesteofibrous dysplasia, Adamantinoma, Tibia, Diaphysis, Histopathology, Extended intralesional curettage, Case report

## Abstract

**Introduction:**

Giant cell tumor (GCT) is a benign bone tumor typically seen in epiphysis or metaphysis of mature long bones. Multiple large multinucleated giant cells dispersed among mononuclear spindle cells and monocytes constitute characteristic histological background of GCT of bone (GCTB).

**Case presentation:**

A 15-year-old girl was admitted to our hospital with the complaint of pain and swelling in the left leg with difficulty in walking for 2 years. On X-ray of the left leg, osteolytic, expansile, eccentric lesion with sclerotic bone margin on the diaphysis of the tibia was seen suggesting oesteofibrous dysplasia. MRI demonstrated findings compatible with adamantinoma. The subsequent histology report was rather surprising, consistent with giant cell tumor of the bone. Extended intralesional curettage was done with the help of a high-speed burr followed by chemical cauterization and bone grafting. The patient was followed up for 2 years. The patient could walk normally without assistance or any signs of a recurrence.

**Discussion:**

GCTB commonly affects people in their third and fourth decades of life and involves epiphysis of the long bone, but this is a case of diaphyseal GCT, at an age of 15 years. It is challenging to diagnose GCT, if present in an unusual location, unless confirmed by histopathological examinations.

**Conclusion:**

A multi-disciplinary approach is required to correctly reach the diagnosis of GCT when it happens to be in an uncommon location(s). Early diagnosis with appropriate treatment and long-term follow-up is mandatory for the successful outcome of the treatment.

## Highlights

• GCTB commonly affects people in their third and fourth decades of life and involves epiphysis of the long bone.
• We report an unusual case of a 15-year-old girl diagnosed as a giant cell tumor in the diaphysis of the tibia.
• Early diagnosis, appropriate treatment, and long-term follow-up are essential for a successful treatment outcome.
• Extended intralesional curettage was performed with the help of a high-speed burr followed by chemical cauterization and bone grafting.
• After surgery and close monitoring at regular intervals for 2 years, the patient was able to walk normally without any signs of recurrence.

## 1 Introduction

Giant cell tumors (GCT) are one of the most common benign bone tumors arising from non-bone forming supportive connective tissue of marrow with a network of stromal cells, regularly interspersed with giant cells. They are locally aggressive and potentially malignant tumors. They typically affect young individuals in their third and fourth decades of life, with female predominance [1], [2]. Compared to the western populations, Asians experience noticeably higher incidences of GCT [3]. It is typically found at the distal femur or proximal tibia's metaphyseal or epiphyseal region. The distal radius, ulna, sacrum, metacarpals, and spine, pelvis, are uncommon places where it can be existent [3], [4], [5], [6], [7], [8], [9]. Ninety percent of the tumors are located in the normal epiphyseal position [1]. Rarely, patients with immature skeletons will develop giant cell tumors in the diaphysis of long bones. In the few instances, where GCT appears in a patient with an immature skeleton, the lesion is more likely to be present in the metaphysis [10]. As per study analysis, metaphysis or diaphysis without epiphyseal involvement is seen in only 1.2 % of GCT cases [3], [9], [11].

However, there is some tumor-like Adamantioma and oesteofibrous dysplasia of bones that are commonly encountered in the diaphysis of the tibia [12], [13], [14], [15]. Similarly, giant cell-rich lesion of bone represents a group of morphologically and biologically diverse tumors with innumerable, non-neoplastic, osteoclast-like giant cells common in all of them, for instance, aneurysmal bone cyst, fibrous cortical defect, brown tumor, giant cell reparative granuloma, giant cell-rich osteosarcoma, and chondroblastoma [14]. The key distinguishing feature among these tumors is in their distinctive clinical and radio graphical characteristics and, most importantly, the histological feature of cell types other than the giant cell.

We report a case of a 15-year-old girl who presented to our institution with chronic leg pain and difficulty in walking for 2 years and was later diagnosed to have a giant cell tumor in the diaphysis of the tibia. More interestingly, this case also created a diagnostic dilemma for the clinicians due to its age, location, and radio graphical appearance. As per literature search, diaphyseal giant cell tumor in a skeletally immature patient has only been described thrice in the literature including the last made by el Shamly et al. [16], [17], [18] and probably our case is the fourth to be reported and the eleventh case overall when encasing the adult population case reports and the entire summary is available in Table 1 [11], [19], [20], [21], [22]. Considering this, it is crucial to be aware of the rare existence of giant cell tumors in areas other than the epiphysis. If not, we might overlook a few.

**Table 1:** Summarizing literature review of the giant cell tumor involving the diaphysis of both skeletally mature and immature patient.

Author	Geographic region	Age and sex	Anatomical location	Biopsy (+/−) and type (FNA, incisional)	Procedure done	Associated factors	Outcome	Follow-up period
Skeletally immature								
Our case	Bangladesh	15 year old female	Diaphysis of the tibia.	Incisional	Extended intralesional curettage followed by chemical cauterization and bone grafting	None	Good	2 years and ongoing.
El shamly et al. 2022 [16]	Rwanda	15 years male	Diaphysis of the radius with multiple relapses	+/incisional	En-bloc resection and ulnar centralization	None	Good	2 years 2 months
Patel et al. 2015 [17]	India	15 years female	Diaphysis of the ulna	+/incisional	Resection + bone graft	None	Good	2 years
Visscher et al. 1988 [33]	US	7 months male	Diaphysis of the ulna	+/not specified	En-bloc resection and fibular graft	None	Good	1 year 3 months

Skeletally mature								
Sandeep et al. 2008 [19]	India	35 years female	Diaphysis of the radius	+/FNA	Resection and centralization of ulnar	None	Good	2 years
Binesh et al. 2012 [20]	Iran	18 year female						
Fain et al. 1993 [11]	US	21 years female	Diaphysis of the tibia	+/not specified	Curettage and bone graft	None	Recurrence after 6 years	4 years
		27 years female	Diaphysis of the tibia	+/not specified	Curettage	None		26 years
		37 years female	Meta-diaphysis of the fibula	+/not specified	En-bloc resection	None		5 years
Darioush et al. 2013 [21]	Iran	46 years female	Meta-diaphysis of the femur	+/incisional biopsy	Curettage and bone cement	None	Good	2 years
Wilkerson et al. 1969 [22]	US	27 years female	Diaphysis of the tibia	–	None	–	–	

## 2 Method

This work has been reported in the line with SCARE 2020 CRITERIA [23].

## 3 Case presentation

A 15-year-old girl came to our hospital when she started experiencing left leg pain while walking to school from her home on a daily basis which was initially gradual in onset, dull aching, intermittent and localized on the anterolateral aspect of the middle part of the left leg for one and a half year. The pain was aggravated by movement and relieved by analgesics. She described the trauma of falling to the ground a year prior, after which her pain increased from mild to severe, getting worse at night. She also noticed swelling in the same region for the last 1 year which is gradually increasing in size. She could move around only with the aid of a crutch. She had no history of fever, anorexia, weight loss, cough, hemoptysis, or any other constitutional symptoms. Her milestones of development are within the normal range. A general examination revealed no abnormality. Her vitals were normal with pulse = 76 bpm, blood pressure = 100/60 mmHg and respiratory rate = 17/min. On loco-regional examination, there was an oval tender swelling on the anterolateral aspect of the middle part of the left leg, measuring 6 × 7 cm, the temperature of which was raised with firm consistency, smooth surface, ill-defined margin, free to overlying skin but fixed to the underlying structure with no regional lymphadenopathy and intact distal neurovascular status. There was no presence of any discharging sinus or any muscle wasting around that area. Gait was antalgic but the range of motion of knee and ankle joints were within normal limit. Systemic examination revealed no abnormality.

Her initial laboratory investigations were done. On complete blood count, her Hb = 12.6 g/dL, WBC count13630/cu.mm, C- reactive protein was 2.16 mg/L. Her Parathyroid Hormone level = 120 pg/mL, S. calcium = 9.6 mg/dL, S.TSH = 1.22 μIU/mL, S. Inorganic phosphate = 2.5 mg/dL, S.LDH = 198 U/L, Alkaline Phosphatase = 86 U/L were within normal range and Mantoux test was negative. X-ray of chest posterior-anterior view revealed a normal study.

**Fig. 1:**
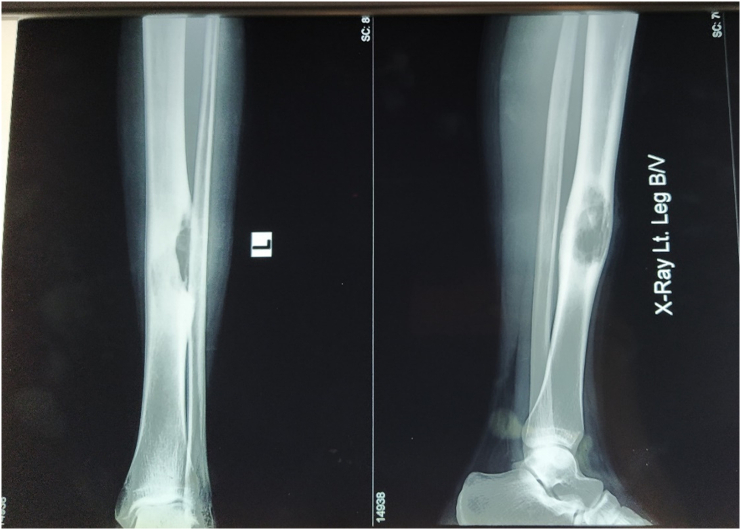
X-ray of left leg showing osteolytic, expansile, eccentric lesion with sclerotic bone margin on the diaphysis of tibia.

X-ray of the left leg as shown in Fig. 1 revealed an osteolytic, expansile, eccentric lesion with sclerotic bone margin on the diaphysis of the tibia suggesting osteofibrous dysplasia. Bone scintigraphy revealed an osteoblastic lesion involving the mid-shaft of the left tibia, compatible with the primary neoplastic bone tumor.

**Fig. 2:**
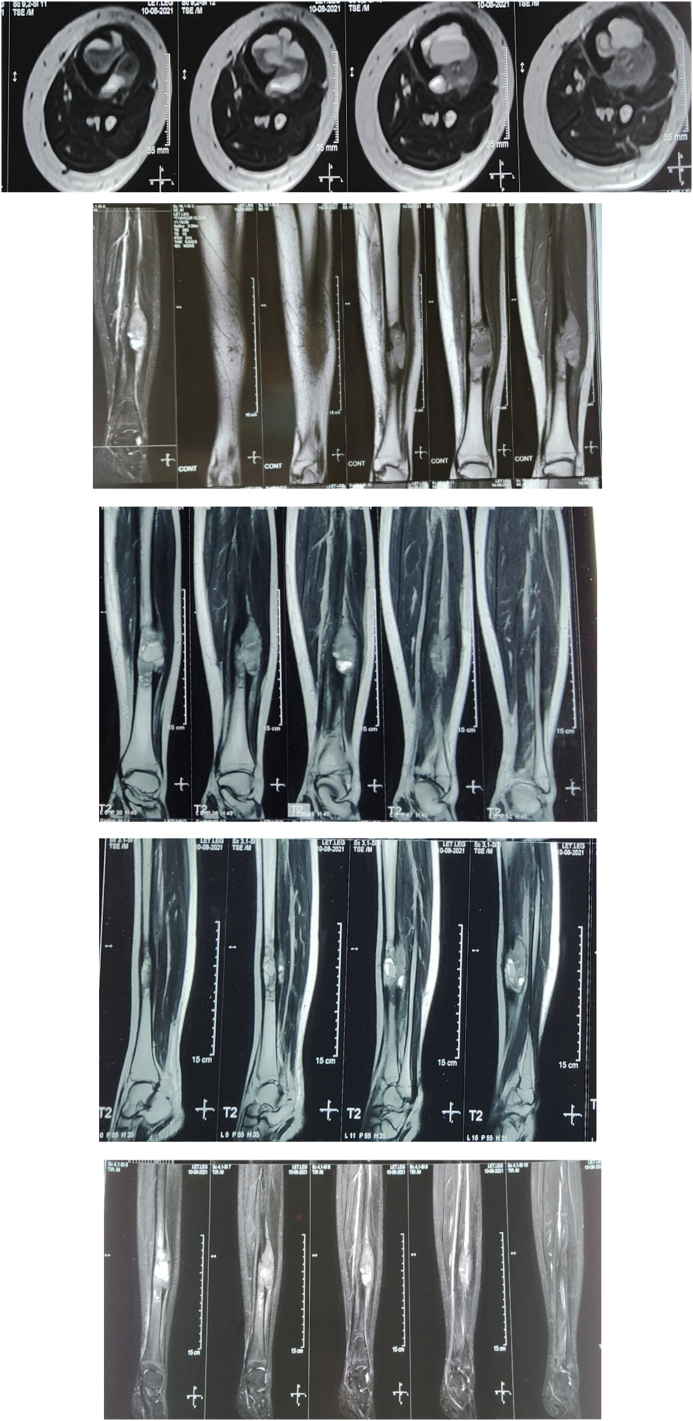
MRI of left tibia showing revealed mixed type of altered signal intensity, expansible lobulated cortical lesion in lower diaphysis of left tibia, showing heterogeneously hypo signal intensity on T1WI and heterogeneously hyper signal intensity on T2WI and STIR images with internal fluid level. The lesion displaced the adjacent muscle. After IV contrast, heterogeneous contrast was seen in the lesion. Subcutaneous soft tissue shows normal signal intensity without focal lesion.

MRI of the left leg with different planes is shown in Fig. 2 which revealed a mixed type of altered signal intensity, expansible lobulated cortical lesion in the lower diaphysis of the left tibia, showing heterogeneously hypo signal intensity on T1WI and heterogeneously hyper signal intensity on T2WI and STIR images with internal fluid level. The lesion displaced the adjacent muscle. After IV contrast, heterogeneous contrast was seen in the lesion. Subcutaneous soft tissue showed normal signal intensity without focal lesion. The findings on MRI were suggestive of adamantinoma in the diaphysis of the left tibia. The patient came from a low-socioeconomic background and could not afford two cross-sectional imaging, so a CT scan was not performed.

**Fig. 3:**
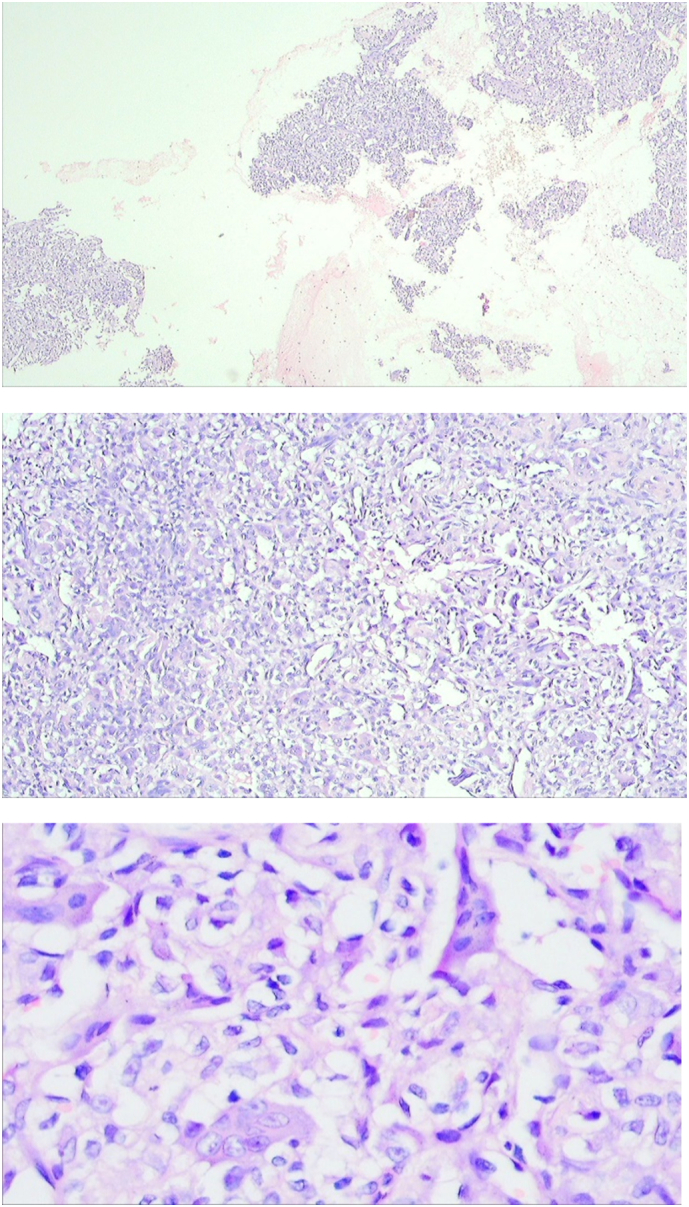
Tissue biopsy from left tibia on H&E staining (on ×120, ×220, ×440 magnification respectively), showed many multinucleated giant cells dispersed with mononuclear cells without presence of any cystic structure compatible with giant cell tumor of bone.

A core-needle biopsy of tissue from the left tibia was performed and sent for histopathological examination. The finding was quite surprising to us as it revealed the presence of a large number uniformly distributed multinucleated giant cells interspersed with sheets of round to oval mononuclear stromal cells without the presence of any cystic structure compatible with giant cell tumor of bone as shown in Fig. 3.

**Fig. 4:**
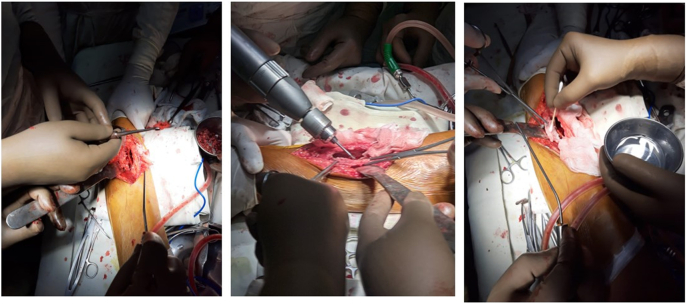
Per-operative picture showing curettage by high speed burr and chemical cauterization with 5 % phenol and 70 % alcohol.

**Fig. 5:**
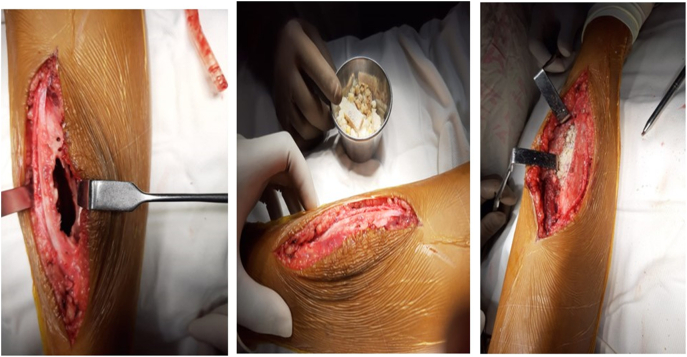
Per-operative picture showing tumor cavity after curettage and tumor cavity filled by autogenic and allogenic bone graft.

Therefore, we decided to go for the operative procedure. All the pre-anesthetic workup was completed. Under spinal anesthesia, exploration and curettage were done by high-speed burr followed by chemical cauterization with 5 % phenol and 70 % alcohol followed by H₂O₂, shown in Fig. 4. The tumor cavity was filled with autogenous and allogenic bone grafts as shown in Fig. 5.

**Fig. 6:**
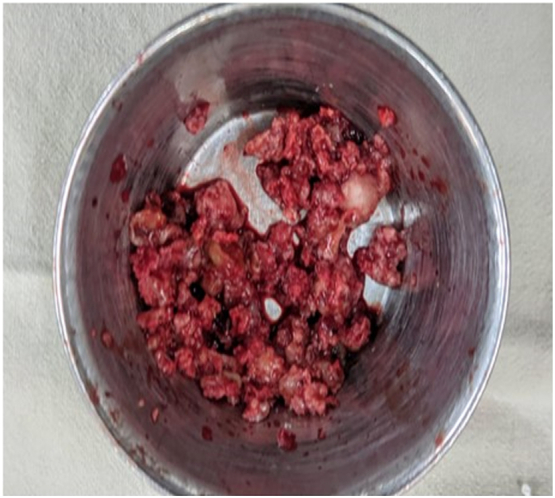
Per-operative picture of reddish brown curetted material. (For interpretation of the references to color in this figure legend, the reader is referred to the web version of this article.)

**Fig. 7:**
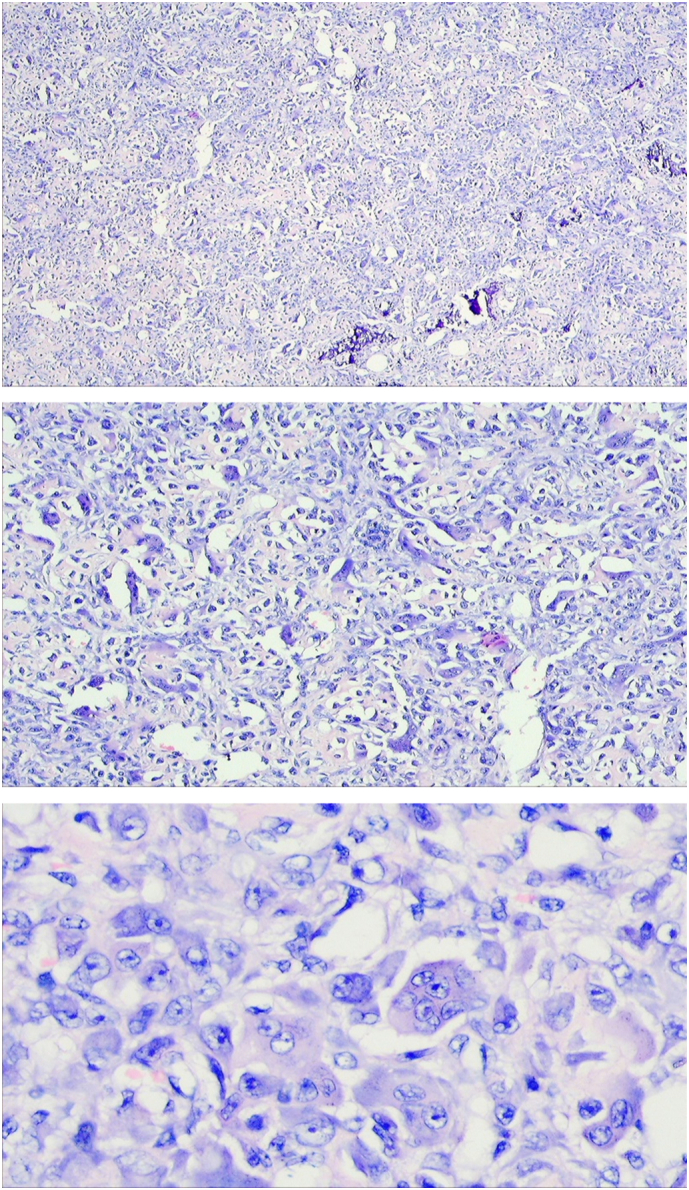
Scattered multinucleated osteoclast like giant cells with round to oval mononuclear stromal cells demonstrating high mitotic figures. Foci of osteoid formation were present with some of the fragments suggestive of old hematoma with features of organization. There were no cystic and necrotic areas.

Reddish brown fleshy mass was found per operatively as shown in Fig. 6 which was sent for histopathological examination which revealed scattered multinucleated osteoclast-like giant cells with round to oval mononuclear stromal cells demonstrating mitotic figures. Foci of osteoid formation were present with some of the fragments suggestive of old hematoma with features of an organization. There were no cystic and necrotic areas. It has been shown in Fig. 7. So the diagnosis of giant cell tumor of the diaphysis of the tibia was confirmed. The postoperative period was uneventful. On the 1st POD, sitting up, breathing exercise, and ankle pump exercise were advised followed by drain tube off, muscle strengthening exercise (Quadriceps, Hamstring), and joint mobilizing exercise on 2nd POD. After removal of the stitch on 14th POD, non-weight bearing for 1st 6 weeks, then toe touching followed by partial weight bearing was allowed for next 6 weeks and full weight bearing was allowed after 12 weeks.

**Fig. 8:**
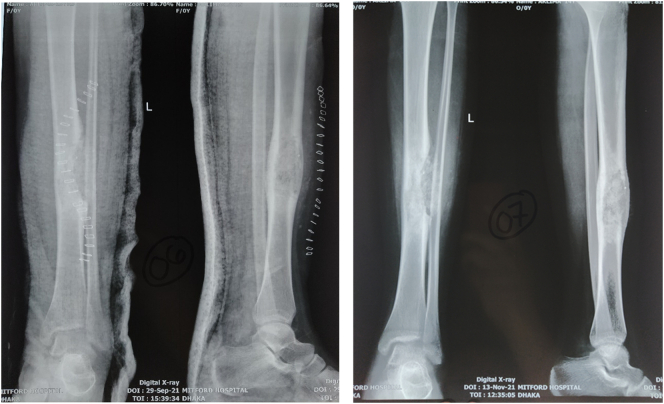
Follow up X-ray on 2nd post-operative day and one and half month after surgery.

**Fig. 9:**
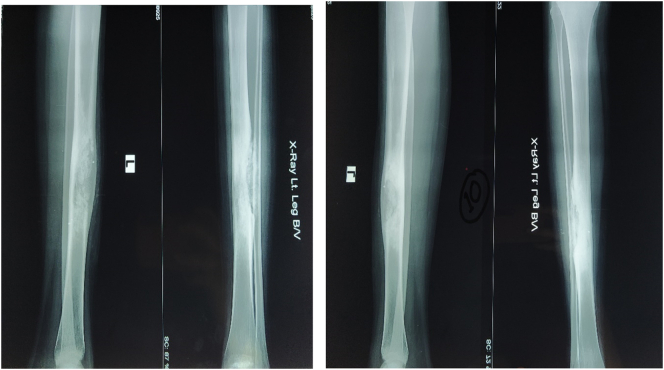
Follow-up X-ray taken after 6 months and 1 year.

**Fig. 10:**
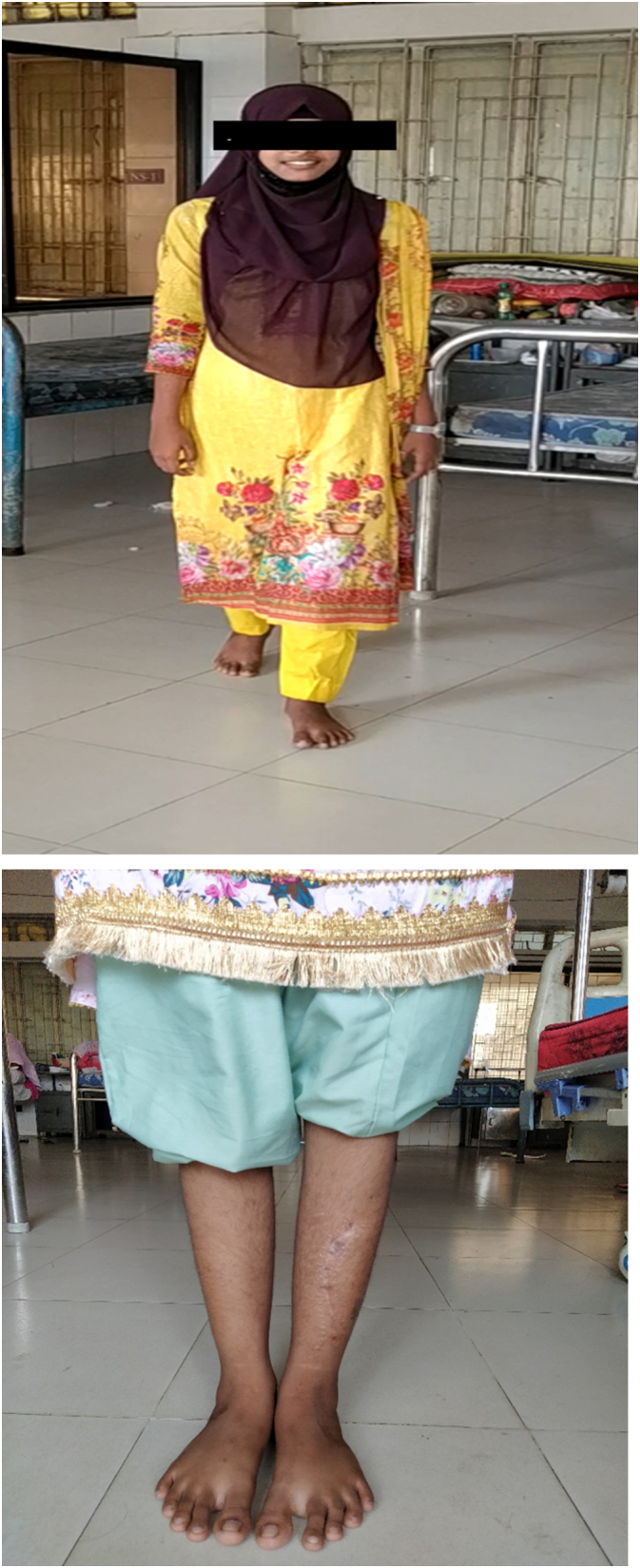
Clinical picture taken 2 year after surgery.

The patient was evaluated after one and half months, 3 months, 6 months, 1 year and 2 year of surgery (as shown in Fig. 8, Fig. 9). It was found that there weren't any complaints and the patient could walk normally without a walking aid. Clinical and radiological evaluation showed neither signs of recurrence nor prognosis to worse condition(s). However, as planned, the patient will be evaluated every year (Fig. 10).

## 4 Discussion

A diagnosis of GCT is questionable without epiphyseal involvement. Even if the radiography results are useful, the diagnosis cannot be made with certainty. The gold standard for diagnosis is still histological analysis. As Jaffe has mentioned ‘A bone lesion may be uncharacteristic in all other respects, but if it exhibits the cytological pattern of a giant cell tumor, it should be recognized as a GCT’ [24]. It can be challenging for a pathologist to distinguish metaphyseal and diaphyseal GCT from other lesions.

Giant cell-rich lesion of bone represents a group of morphologically and biologically diverse tumors with innumerable, non-neoplastic, osteoclast-like giant cells common in all of them, for instance, aneurysmal bone cyst, fibrous cortical defect, giant cell lesion of hyperparathyroidism (brown tumor), giant cell reparative granuloma, giant cell-rich osteosarcoma, and chondroblastoma [14]. Similar to GCTs, aneurysmal bone cysts show up in younger age groups as an expanding firm mass. Blood-filled cystic areas divided by fibrous septa are the histological characteristics of a primary or secondary aneurysmal bone cyst. They appear to be a radiolucent expansile cystic, multi-loculated lesion with cortical thinning on x-rays, typically involving the metaphysis [5], [25]. A vascular fibroblastic stroma that is often missing in GCTB in the background of osteoclast-like multinucleated giant cells characterizes brown tumors, which are typically related to hyperparathyroidism. There isn't much information available regarding these tumors because they are so uncommon [19], [25].

Using magnetic resonance imaging (MRI), one may determine how far a tumor has infiltrated the soft tissue and bone marrow. The tumor's appearance on T1-weighted imaging is decreased signal intensity, while tumor appearance on T2-weighted images is increased signal intensity, and shows enhancement on gadolinium-enhanced images. While such findings in MRI are shared not only by GCT but also by other bone tumors like adamantioma and osteofibrous dysplasia. This justifies making a diagnosis only after histologically ruling out other possibilities [13], [26].

GCTB appears dark brown to reddish in color and friable in nature. The histological appearance of GCTB is characterized by the presence of multiple large multinucleated giant cells dispersed in the background of mononuclear spindle cells and monocytes. Similar to the radiographic variability seen in these lesions, the histological appearance can also be extremely diverse and include areas of cystic degeneration, hemorrhage, hemosiderin deposition, and occasional mitotic figures (no atypical mitotic figures), or increased spindle cell stroma. Mononuclear spindle cells make up the GCTB's malignant cells. The mononuclear stroma cells are thought to be principally responsible for the formation of the giant cells via the receptor activator nuclear factor K—B ligand (RANKL) [25].

Surgery is still the gold standard therapy for this pathology. The surgical options available today range from intralesional curettage with bone cement or bone grafting, cryotherapy to marginal resection, extensive local resection, or en-bloc resection. However, there is a paucity of trials evaluating the different options [3], [25]. As there is a wide spectrum of treatment modalities with variable recurrence, therefore, no option can be chosen as the best one. An aggressive local tumor removal procedure called extended intralesional curettage is augmented additionally by mechanical, thermal, or chemical adjuvant therapy. The tumor kill zone is believed to be extended by the use of adjuvant therapies several millimeters beyond the limits of mechanical curettage. A cortical window is made to allow the visualization of the lesion, and curettage of the visible portion of the lesion is done. Subsequently, a high-speed burr is mechanically introduced to extend the boundaries of the resected lesion. Several adjunct therapies, including phenol/alcohol, liquid nitrogen, hydrogen peroxide, and argon beam have been explored, however, none has shown superior outcomes [1], [25], [27]. To fill osseous cavities left by excision, bone cement or bone graft (allograft, synthetic composites, etc.) have all been utilized. Some authors have suggested that an allograft buffer inhibits the heat necrosis of the chondrocytes caused by the exothermic reaction of the bone cement [28], [29], [30]. An effective treatment strategy has been demonstrated to be local control, with extensive intralesional excision. Wide excision, which was once the primary treatment for GCTB, is now often only used in situations when there has been a local recurrence or when the tumor has any significant extraosseous extension. The most crucial part of this treatment is complete tumor removal, which should be carried out through an adequate cortical window that provides visualization of the entire defect. If there has been significant bone damage or if less invasive treatments have failed for the patient, radical resection is usually needed [25], [27].

Besides that, adamantinoma, osteofibrous dysplasia, and osteofibrous dysplasia-like adamantinoma are common related disorders that mostly affect diaphysis of the tibia [20]. Their radiological and histological characteristics fall into a continuum, making distinction very difficult. They are extremely similar radiological lesions that most frequently develop anteriorly, in the mid-diaphysis of the tibia [13], [26]. A malignant biphasic tumor called an adamantinoma can have some morphological patterns, most frequently clusters of epithelial cells, surrounded by a band of spindle osteofibrous component(s). A striking feature of this adamantioma is its predilection for involvement of the mid-shaft of the tibia, which accounts for about 85 % of all cases [18]. Among other long bones, fibula and ulna are rarely affected. Radiological imaging of adamantioma typically shows a mid-diaphyseal osteolytic, eccentric, expansile lesion in the tibia which is medullary in location. It has a characteristic “soap-bubble” appearance due to the presence of multifocal radiolucencies surrounded by ring-shaped densities [13].

A benign fibro-osseous disease that causes deformities in children called osteofibrous dysplasia has a significant propensity to affect the midshaft of the tibia, either with or without the involvement of the fibula. In the series of 80 cases reported by Park et al., 77 involved the tibia and three the fibula. In nine of the cases, both the tibia and fibula were involved on the ipsilateral side. Other reported sites of involvement are the ulna and the radius [31]. Pseudo arthrosis and a bowed tibia are both potential consequences. It initially appears radiologically as an intra-cortical lesion that is radiolucent, fairly well marginated with marginal sclerosis. It may also display a “ground-glass” appearance. The osteoid osteoma, Brodie's abscess, and osteoblastoma are among the possible radiological differential diagnosis. Lately, the third group of cases with clinical and radiological features similar to those of osteofibrous dysplasia has demonstrated more overt strands of epithelial cells within a fibro-osseous background and has been categorized as “differentiated”, or “osteofibrous dysplasia- like adamantinoma”. OFD-like adamantinoma and osteofibrous dysplasia have similar histopathological patterns, thus, pathologists must be aware to perform immunohistochemical staining for keratin particularly when the histopathological features of osteofibrous dysplasia show small nests of epithelial tumor cells within the fibrous stroma [13]. OFD tends to show a diffused cytokeratin immunostaining. In contrast, OFD-A shows focal staining of small nests of epithelial cells. The small nests of epithelial cells and individual keratin-positive cells in the stroma are the characteristic feature of OFD-like adamantinoma [13], [26]. Complete involvement of the medullary cavity is almost always seen in an adamantinoma. In contrast to OFD and OFD-like adamantinoma, intra-medullary involvement is minimal or absent [32]. Consequently, treatment of patients with a diagnosis of OFD or differentiated adamantinoma tends to be conservative while patients with classic adamantinoma are treated more aggressively. Therefore, clinicians should be aware of such tumors and their common and uncommon location(s). Following the treatment, assessment and monitoring of the patient, with serial radiographs of the chest and the site of involvement, along with thorough physical examinations play a pivotal role in detecting incidents of recurrence. However, tumor recurrences have been detected many years after the initial treatment, which is suggestive of at least a 5-year close follow-up.

## 5 Conclusion

Though GCTB commonly involves epiphysis of the long bone, it may sometimes also involve unusual locations like the diaphysis of the long bone. Besides, radiological examination, histopathology from tissue biopsy is the court of appeal for the confirmatory diagnosis and plan the further management accordingly. Early diagnosis, appropriate treatment, and long-term follow-up are essential for a successful treatment outcome. Furthermore, it is imperative to educate patients about the presentation of local invasion or metastasis, so that they can pursue early medical advice. In our case, the patient had no complaints and could walk normally without any signs of recurrence, after surgery and meticulous evaluation at regular intervals for 2 years. However, as planned, the patient will be evaluated every year.

## Consent

Written informed consent was obtained from the patient for publication of this case report and accompanying images. A copy of the written consent is available for review by the Editor-in-Chief of this journal on request.

## Ethical approval

Nothing to declare.

## Funding

This research did not receive any grant from funding agencies in the public, commercial, and non-profit organization.

## Author contribution

HM was the chief surgeon and performed the entire surgery with his team.

PY conceived, designed and took entire responsibility for publishing this study under supervision of HM

PY and SY helped in drafting and editing the manuscript.

HM and MK reviewed the manuscript.

All authors provided intellectual input to the study and approved the final version of the Manuscript.

## Guarantor

Prashant Yadav and MD Hasan Masud accept full responsibility for the work and/or the conduct of the study, had access to data, and controlled the decision to publish.

## Research registration number

Not applicable.

## Provenance and peer review

Not commissioned, externally peer-reviewed.

## Declaration of competing interest

None to declare.
